# Effects of RAFT Agent on the Chloromethylstyrene Polymerizations in a Simultaneous Radiation Grafting System

**DOI:** 10.3390/polym9080307

**Published:** 2017-07-26

**Authors:** Jinhua Chen, Noriaki Seko

**Affiliations:** Department of Advanced Functional Materials Research, Takasaki Advanced Radiation Research Institute, National Institutes for Quantum and Radiological Science and Technology (QST), 1233 Watanuki-Machi, Takasaki, Gunma 370-1292, Japan; seko.noriaki@qst.go.jp

**Keywords:** reversible addition-fragmentation chain transfer (RAFT), radiation, grafting copolymers, chloromethylstyrene, ethylene-tetrafluoroethylene copolymer (ETFE)

## Abstract

Reversible addition-fragmentation chain transfer (RAFT) agent was added into a simultaneous radiation grafting system and its effects on graft polymerization and homopolymerization were investigated. Chloromethylstyrene (CMS) was graft polymerized onto ethylene-tetrafluoroethylene copolymer (ETFE) films under γ-ray sources via simultaneous irradiation. The non-grafted poly(CMS) in the grafted films were extracted by xylene at 120 °C. The poly(CMS) was characterized by NMR and GPC instruments. Addition of the RAFT agent suppressed both graft polymerization and homopolymerization. However, under a high concentration of RAFT agent, the homopolymerization in the monomer solution could occur through a typical RAFT polymerization while polymerization in the ETFE films proceeded via RAFT and conventional radical polymerization, resulting in poly(CMS) in the ETFE films with molecular weight dispersity higher than 1.0 but lower than that without RAFT agent. Furthermore, it was found that the molecular weight of the poly(CMS) in the ETFE films was several times higher than that of the poly(CMS) in the monomer solution.

## 1. Introduction

Reversible addition fragmentation chain transfer (RAFT) polymerization is a reversible deactivation radical polymerization (RDRP) that can provide living characteristics for precise synthesis of polymers with well-defined constructs and designed molecular weights [1,2,3,4]. Thiocarbonylthio compounds, having the general structure S=C(Z)SR, where the R and Z are leaving group and activating group, respectively, are usually used as RAFT agents. RAFT polymerization is a dynamic equilibrium between active radicals (propagating radicals) and dormant radicals via the reversible addition-fragmentation of the thiocarbonylthio groups. The initial radicals can be created from thermal decomposition of organic peroxides and azo compounds [5], reduction of hydrogen peroxide [6], thermal treatment [7], photolysis by UV or visible light [8], and ionizing irradiation [9,10,11,12,13].

On the other hand, radiation grafting can introduce new properties, such as proton-conductivity, ion-exchange, and catalytic ability, into existing polymer films while maintaining their inherent characteristics. To date, radiation grafting has been widely employed for the development of polymer electrolyte membranes for fuel cells and absorbents for recovery of metal ions [14,15].

Radiation grafting can be carried out either by simultaneous or pre-irradiation methods [14,15,16,17,18,19,20,21,22,23,24,25,26,27,28]. In the former, both the monomer and base films are irradiated simultaneously, and the grafting was carried out under the irradiation environments [19,20], while in the latter, the base films are irradiated initially and the grafting is performed after irradiation [21,22,23]. Homopolymerization in the monomer solution is a solution-phase reaction that strongly affects graft polymerization and lowers monomer utilization. On the contrary, graft polymerization is a solid-phase reaction that occurs from the surface to the interior of the films as the monomer diffuses, according to the so-called grafting front mechanism [24].

We tried to introduce RAFT polymerization into radiation grafting to precisely control the grafting process and to suppress homopolymerization as much as possible. In this study, the RAFT agent, 2-cyano-2-propyl benzodithioate (CPB), was added to the simultaneous irradiation grafting systems, in which chloromethylstyrene (CMS) was grafted onto ethylene-tetrafluoroethylene copolymer (ETFE) films. The resulting ETFE-*g*-poly(CMS) films are versatile materials because various functional groups could be introduced through chemical reaction with the active chloromethyl groups. For example, anion-exchange membranes as electrolyte and separator for fuel cells had been developed by quaternization of the ETFE-*g*-poly(CMS) films [25,26,27].

The molecular weight of the graft chains in the solid films has rarely been analyzed. This is due to the difficulty in separating the graft chains without degradation from the base films. Recently, a selective decomposition method was reported to separate the polystyrene graft chains from the cellulose-*g*-polystyrene materials, where the cellulose were hydrolyzed and the molecular weight of the graft chains were successfully determined by GPC instrument [29,30,31]. Such methods can be extended to polymer grafted inorganic particles, where the particles can be removed by acid treatment [32,33,34,35,36]. However, the fluororesin ETFE film is a thermally and chemically stable material, and it is difficult to selectively decompose the ETFE chains without the deterioration of the graft chains. More recently, Enomoto et al. separated hydrophilic graft chains from hydrophobic base materials by immersing the ETFE-*g*-poly(styrene sulfonic acid) in hot water, in which the high interface stress between the two opposite phases prompted the separation [37].

In this study, we found that a considerable amount of poly(CMS) could be extracted from the grafted ETFE films by xylene at 120 °C. The extracted poly(CMS) may be the homopolymer (non-grafted poly(CMS)) formed in the interior of the ETFE films. Even then, they were polymerized under the same environments as the grafted poly(CMS) chains, and was expected to have the same properties, such as molecular weight and dispersity.

By analyzing the molecular weight of both the poly(CMS) extracted from the grafted films and the poly(CMS) formed in the monomer solution, the effects of RAFT agent on the simultaneous radiation grafting were established. Furthermore, the effects of irradiation dose, irradiation dose rate and mole ratio of [RAFT]/[CMS], on the polymerization in the ETFE films and in the monomer solutions were discussed in detail.

## 2. Materials and Methods

### 2.1. Materials

The poly(ethylene-*co*-tetrafluoroethylene) (ETFE) films (Aflex, 25 μm in thickness) were purchased from Asahi Glass Co., Ltd., Tokyo, Japan, and the chloromethylstyrene (CMS) monomer was purchased from AGC Seimi Chemical Co., Ltd., Chigasaki, Japan. The received yellow CMS monomer was purified by alternately washing with sodium hydroxide aqueous solution and water to remove the *tert*-butylcatechol inhibitor. The purified transparent CMS monomer was kept in a glass bottle containing anhydrous MgSO_4_ at 5 °C. The RAFT agent, 2-cyano-2-propyl benzodithioate (CPB), was purchased from Sigma-Aldrich Co., Ltd., Tokyo, Japan and was used as received. The chemical reagents such as acetone, toluene, xylene, as well as tetrahydrofuran (THF, HPLC reagent) and d-chloroform (CDCl_3_) were purchased from Wako Pure Chemical Industries, Ltd., Tokyo, Japan and were used without any further treatment.

### 2.2. Grafting of CMS onto ETFE Films

The grafting of CMS onto ETFE films was carried out by a simultaneous radiation grafting method [19], where the ETFE films (2 cm × 3 cm in area) were completely immersed in the monomer solution in a reaction tube for γ-ray irradiation. The monomer solution consisted of CMS monomer (0.02 mol), acetone solvent (6.0 g), and the designed amount of RAFT agent. Herein, the monomer solution was bubbled with nitrogen gas for 5 min and then sealed by a rubber stopper. The sealed tube was placed in a cobalt-60 irradiation facility for γ-ray irradiation at room temperature. After irradiation, the films were taken out from the tube and carefully washed with toluene several times at room temperature to remove the homopolymer and residual monomer on its surface. The obtained ETFE-*g*-poly(CMS) films were dried at 60 °C for 24 h. The degree of grafting (GD) was calculated using Equation (1).

Degree of grafting (GD, %) = (*W*_g_ − *W*_o_)/*W*_o_ × 100 (1)

where *W*_o_ and *W*_g_ are the dry weights of the films before and after irradiation, respectively. Herein, all of the poly(CMS) formed in the ETFE films, with and without graft bonds, were regarded as the graft chains for the calculation of the degree of grafting.

### 2.3. GPC and NMR Characterizations

The homopolymers in the irradiated monomer solutions were analyzed by GPC and NMR. After irradiation, the acetone in the irradiated monomer solution was evaporated using a rotary evaporator at 40 °C for 10 min. About 5 μL of the acetone-free solution was directly injected into the GPC system to record the GPC curve. The GPC system (Hitachi High-Tech Sci. Corp., Tokyo, Japan) was equipped with a UV-detector (254 nm), a refractive index detector and two columns (Shodex, GPC KF-805L, Tokyo, Japan). The columns were controlled at 40 °C, and the THF eluent was fixed at a flow rate of 1.0 mL/min. The acetone-free solution was also diluted with CDCl_3_ containing small amount of tetramethylsilane. The tetramethylsilane was used as the internal standard for calibrating the chemical shift of the NMR spectra. About 0.5 mL of the resultant solution was transferred in an NMR tube to record the ^1^H NMR spectra using a Bruker AV 300 MHz spectrometer at room temperature.

### 2.4. Poly(CMS) Extraction and Characterization

After careful washing with toluene at room temperature, the ETFE-*g*-poly(CMS) films were immersed in xylene solvent at 120 °C. Under the immersion, the weight of the grafted films was reduced and the non-grafted poly(CMS) in the interior of the grafted films was extracted. The weight loss (%) after xylene immersion was calculated using Equation (2).

Weight loss (%) = (*W*_g_ − *W*_i_)/(*W*_g_ × GD/(100 + GD)) × 100% (2)

where *W*_g_ and *W*_i_ are the dry weights of the grafted films before and after xylene immersion, respectively, and GD is the degree of grafting. The dry weight was obtained after drying the corresponding samples in a vacuum oven at 80 °C for 6 h. The xylene absorption of the grafted films was calculated using Equation (3).

Xylene absorption (%) = (*W*_x_ − *W*_i_)/*W*_i_ × 100% (3)

where *W*_x_ is the weight of the xylene-absorbed films and W_i_ is the dry weight of the grafted films as described in Equation (2). The *W*_x_ was obtained by weighing the films taken out from xylene after wiping off the excess xylene on the surface.

About 5 µL of the resultant xylene immersion solution was directly injected into the GPC instrument for analysis. The xylene in the final immersion solution was removed using a rotary evaporator at 60 °C for 60 min. The residue was dissolved in CDCl_3_ for NMR measurements.

## 3. Results and Discussion

### 3.1. Simultaneous Radiation Grafting of CMS onto ETFE Films

**Scheme 1:**
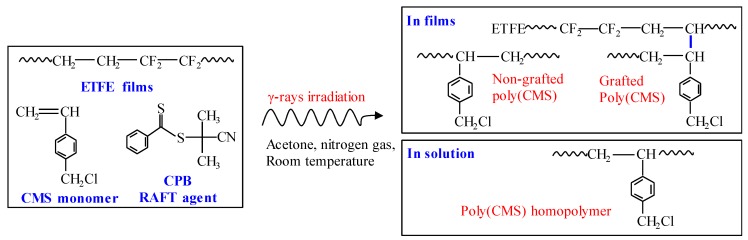
Process for the simultaneous radiation grafting of chloromethylstyrene (CMS) onto ethylene-tetrafluoroethylene copolymer (ETFE) films.

Scheme 1 shows the process for the simultaneous radiation grafting of CMS monomer onto ETFE films. The ETFE film and CMS monomer were simultaneously irradiated by γ-rays at room temperature. During irradiation, radicals were generated on the ETFE chains as well as on CMS molecules. The radicals on ETFE chains initiated graft polymerization, whereas the radicals on CMS molecules initiated the homopolymerization. Thus, the homopolymerization may occur in both the monomer solution and the interior of the ETFE films. Although the poly(CMS) homopolymer in the ETFE films do not have graft bonds to the ETFE chains, they are immobilized in the films and are difficult to remove with toluene washing at room temperature.

In this study, all poly(CMS) generated in the ETFE films, with or without graft bonds, was defined as graft chains, and the weight increase of the films after graft polymerization was used to calculate the degree of grafting (see Equation (1)). On the contrary, the term homopolymerization was used only for the reaction in the monomer solution.

**Scheme 2:**
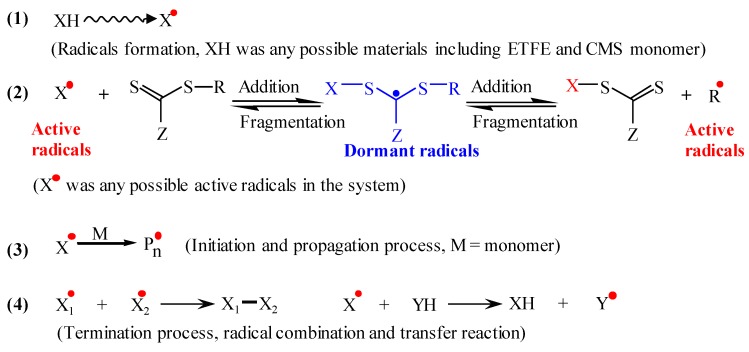
Possible reactions in the simultaneous radiation grafting system.

The possible reactions for the RAFT polymerization in the simultaneous radiation grafting system are given in Scheme 2 [8,18]. During irradiation, active radicals form on the chemical materials due to bond scission or hydrogen abstraction (formula 1 in Scheme 2). In the presence of RAFT agent, the active radicals quickly add to the RAFT agent, resulting in the formation of dormant radicals, which would fragment again to generate the active radicals (formula 2 in Scheme 2). On the other hand, the active radicals can also initiate monomer polymerization (formula 3 in Scheme 2). Here, the shorter propagating groups on the dormant radicals are considered to be more easily to fragment for subsequent polymerization [38,39]. Therefore, the RAFT agent strongly affects polymerization, and lowers the molecular weight dispersity of the resulting polymers. Furthermore, the reversible addition-fragmentation reaction in formula 2 is an equilibrium process, which keeps the lifetime of radicals longer. When there are adequate amounts of RAFT agents, the formulas 2 and 3 repeat alternately until the monomer was completely consumed. Therefore, the molecular weights of the propagating chains increase with polymerization time. However, in a practical system, radical combination and transfer reaction also occur as shown in formula 4. Here, the active radicals in formulas 2~4 include all the radicals, such as the new radicals generated by irradiation, the propagating chains or macromolecule radicals, and the leaving groups fragmented from the RAFT agent and dormant radicals.

### 3.2. Homopolymerization in the Monomer Solution

**Figure 1:**
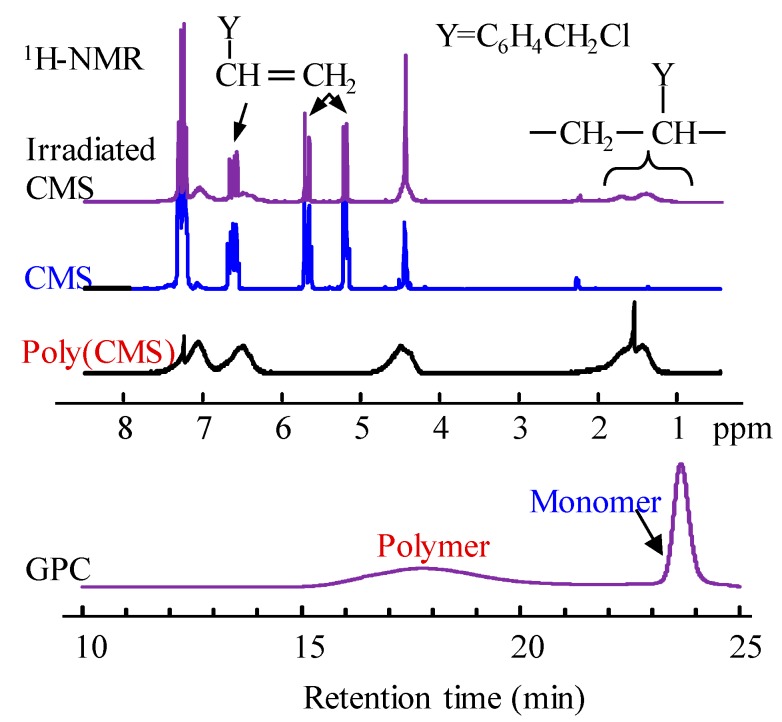
NMR spectra for irradiated CMS monomer, new CMS monomer and commercial poly(CMS), and GPC curve for the irradiated CMS monomer. The irradiated CMS monomer solution was obtained from the grafting system irradiated with a total dose of 50 kGy at a dose rate of 1.0 kGy/h.

The monomer solution obtained from the grafting system after irradiation, as well as the CMS monomer and commercial poly(CMS) were analyzed by NMR. As seen in the NMR spectra (Figure 1), poly(CMS) showed very broad signals due to its high molecular weight. The broad signals around 1.2–2.0 ppm was assigned to the three protons on the polymer main chains (–H_2_CH<). On the contrary, the CMS monomer showed very sharp proton signals. The signals for the three protons on the vinyl moieties (CH_2_=CH–) of the CMS monomer were well-separated and located at 6.7, 5.8, and 5.3 ppm. The irradiated CMS solution obtained from the grafting system showed signals that can be found on the above-mentioned CMS and poly(CMS) spectra, indicating that some of the CMS monomer were homopolymerized under irradiation. Therefore, the homopolymer yield (%) in the monomer solution can be calculated using Equation (4).

Homopolymer yield (%, NMR) = *S*_1.2–2.0_/(3*S*_5.8_ + *S*_1.2–2.0_) × 100% (4)

where *S*_1.2–2.0_ is the total integral area of the peaks in the range of 1.2–2.0 ppm, corresponding to the amount of homopolymer in the solution, and 3*S*_5.8_ is thrice the integral area of the peak around 5.8 ppm, corresponding to the amount of monomer in the solution. Since the amount of the monomer polymerized in the ETFE films was relatively small, it was not included in the calculation.

The monomer solution obtained from the grafting system after irradiation was also analyzed by GPC. As seen in Figure 1, the GPC curve obtained from the refractive index detector showed two peaks, a broad peak around 15.0–20.5 min, corresponding to the homopolymer, and a sharp peak at 23.6 min, corresponding to a molecular weight of 153.5, thus a molar mass of 153.5 g mol^−1^ for the CMS monomer. Therefore, the homopolymer yield (%) in the monomer solution can be calculated by Equation (5).

**Figure 2:**
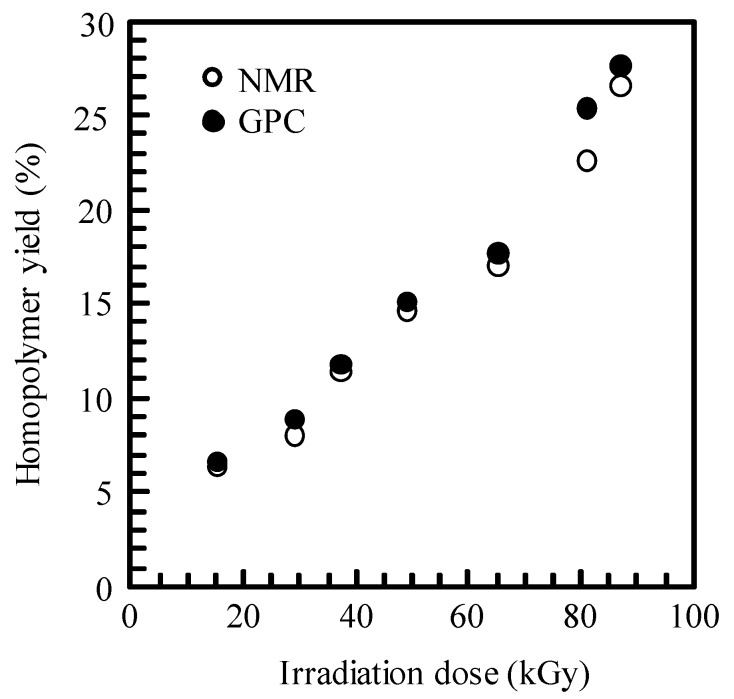
Homopolymer yield in the irradiated monomer solution determined by NMR and GPC methods as a function of the total irradiation dose. Dose rate, 1.0 kGy/h; [RAFT]/[CMS], 0.014; room temperature.

Homopolymer yield (%, GPC) = *S*_p_/(*S*_p_ + *S*_m_) × 100% (5)

where *S*_p_ and *S*_m_ are the integral areas of the polymer and monomer peaks on the GPC curve, respectively. The degrees of homopolymerization determined by NMR and GPC methods were plotted in Figure 2 as a function of irradiation dose. Obviously, the homopolymer yields determined by the two methods were well in agreement with each other. Therefore, the GPC method was used to analyze the homopolymer yield in this work. This was very convenient for the researchers because the molecular weight of the homopolymer and the homopolymer yield could be obtained simultaneously by GPC. As shown in Figure 2, the homopolymer yield linearly increased with the irradiation dose, reflecting a typical character of the RAFT polymerization in the monomer solution under the applied experimental conditions [1,2,3,4].

### 3.3. Characterization of the Graft Chains

To date, the graft chains of the grafted materials were qualitatively well characterized by FTIR, SEM–XRD, TEM, TGA/DSC, XPS, etc. [14,15]. However, quantitative characterization of the graft chains, such as molecular weight and its dispersity, had been rarely reported. This is may possibly be due to the difficulty in separating the graft chains from the base materials for analysis. Even then, the molecular weight and dispersity, which refer to the length and the length distribution of the graft chains, are the most important parameters for the precise design of the high performance graft materials. In some reports the molecular weight of the homopolymer were used to identify the graft chains since they were polymerized in a similar environment [28,29,36,40].

**Figure 3:**
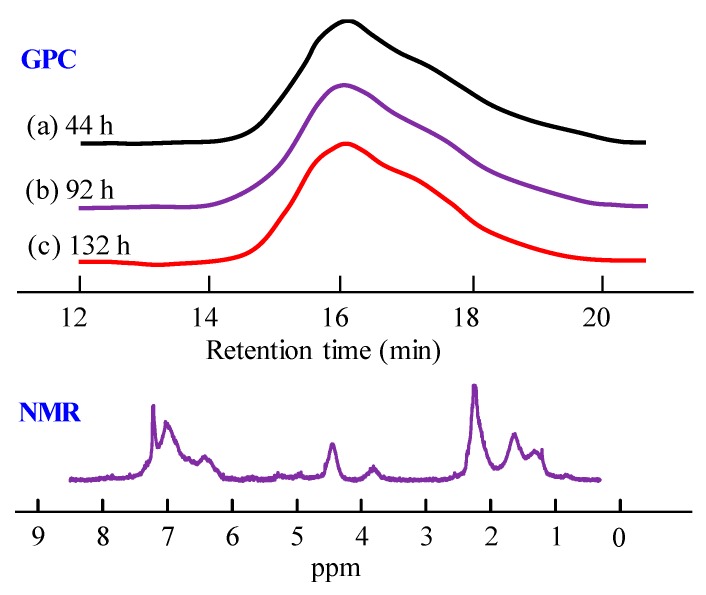
GPC and NMR results of the extracted materials from the ETFE-*g*-poly(CMS) film.

In this study, we immersed the ETFE-*g*-poly(CMS) films in a xylene solvent at a high temperature of 120 °C. A small amount of the immersion solution was taken out periodically for GPC measurement. The final solution after 132 h immersion time was dried by a rotary evaporator and the residue was dissolved in CDCl_3_ for NMR measurement. The GPC and NMR results are shown in Figure 3.

The ^1^H NMR spectra of the residue showed broad signals at 1.2–2.0, 4.0–5.0, and 6.0–8.0 ppm, similar to the signals from poly(CMS), as shown in Figure 1. The strong signals close to 2.3 and 7.2 ppm were assigned to the xylene residue. There were no other signals that originated from the CMS monomer or the ETFE base chains. Therefore, it can be concluded that only the poly(CMS) was extracted from the grafted films and dissolved in the xylene solution during the immersion. As showed in Scheme 1, both the grafted and non-grafted poly(CMS) chains were formed in the interior of the ETFE films under the simultaneous irradiation. In the hot xylene, interface stress occurred between the graft phase and the ETFE base materials due to the different swelling ability in xylene. This interface stress promoted the extraction and gradually separated the non-grafted poly(CMS) from the grafted films.

In addition, as shown in Figure 3, the GPC curves for the samples at 44, 92, and 132 h immersion times were very similar to each other, showing peaks around 16 min retention time, which can be assigned to the poly(CMS). Therefore, the extracted poly(CMS) were stable in hot xylene and did not deteriorate during immersion. The extracted non-grafted poly(CMS) chains were polymerized under the same environments as the grafted ones in the reaction tube, and were expected to have the same properties. Therefore, by analyzing the extracted poly(CMS) the molecular weight and dispersity of the graft chains could be understood. The number average molecular weight (*M_n_*) and dispersity of the extracted poly(CMS) chains calculated from the GPC curves in Figure 3 were about 17,200 ± 600 and 2.14 ± 0.05, respectively.

Furthermore, it was confirmed that the weight of ETFE-*g*-poly(CMS) during immersion decreased. The weight loss was calculated as percent decrease in weight relative to the total weight of poly(CMS) in the ETFE films. It was found that the weight loss (%) after 44 h was 17.5%, and then slowly increased to 19.6% and 22.9% after 92 h and 132 h immersion time, respectively. The poly(CMS) finally remained in the grafted films may be the real graft chains having C–C grafting bonds to the ETFE chains, and was considered difficult to extract without decomposition. High weight loss could be achieved by immersing the grafted films for a very long time but this treatment could also result to degradation of the grafted materials. In addition, the GPC curves of the resulting solution did not show a peak in the polymer region, indicating that the poly(CMS) was completely decomposed under the very long treatment in hot xylene. In this study, the immersion time for the extraction of the grafted materials were carried out for 44 h unless otherwise stated.

On the other hand, the xylene absorption of the grafted film decreased from an initial value of 22.4% to 19.0%, 18.0%, and 14.8% at the above-mentioned immersion periods, respectively. The decreased xylene absorption was due to the loss of the poly(CMS) chains in the grafted materials.

### 3.4. Effect of Total Dose of Irradiation

**Figure 4:**
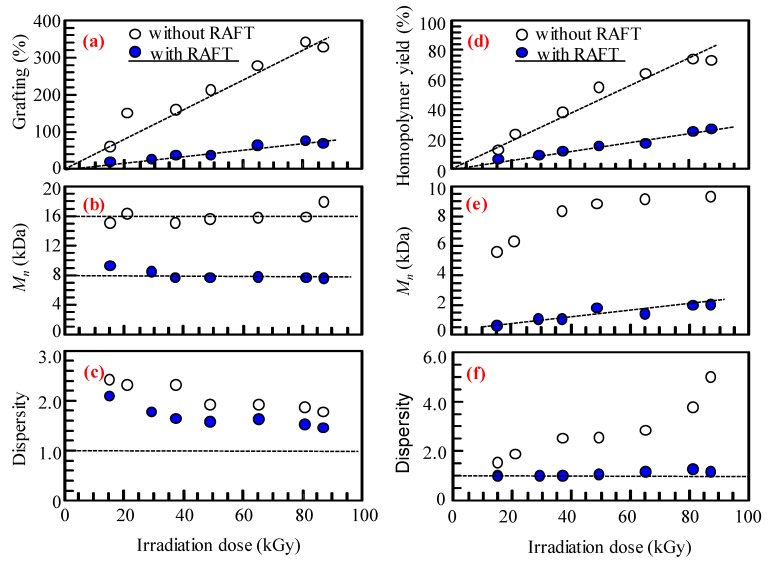
(**a**) Degree of grafting, (**b**) average molecular weight (*M_n_*), (**c**) dispersity of the extracted poly(CMS), and (**d**) homopolymer yield, (**e**) *M_n_*, (**f**) dispersity of the homopolymer in monomer solution as a function of the irradiation dose. Irradiation dose rate, 1.0 kGy/h; [RAFT]/[CMS], 0.014.

The grafting of CMS onto ETFE films was initially investigated at a low dose rate of 1.0 kGy/h. The resulting ETFE films and monomer solutions after irradiation were characterized in terms of degrees of grafting, homopolymer yield, molecular weight, and dispersity, and the results are shown in Figure 4. As shown in Figure 4a, the degree of grafting increased with increasing irradiation dose. However, when the RAFT agent was added, the degree of grafting decreased. For instance, the degrees of grafting were about 75% and 334% after irradiation to a total dose of 88 kGy, with and without RAFT agent, respectively.

The homopolymer yield in the irradiated monomer solution showed a similar tendency to degree of grafting (Figure 4d). That is, the homopolymer yield increased with irradiation dose, and decreased considerably in the presence of RAFT agent. Without RAFT agent, too much of the homopolymers were formed in the solution. With an irradiation dose of 88 kGy, the homopolymer yield exceeded 70% and the grafted films were difficult to take out due to the high viscosity of the monomer solution. On the contrary, addition of RAFT agent suppressed homopolymerization in the solution and even after irradiating to a high irradiation dose of 88 kGy, the homopolymer yield was less than 30%. This phenomena was similar to the addition of metal ions, such as Fe(II) and Cu(II), into the simultaneous grafting systems for inhibiting the homopolymerization in the monomer solution [41,42,43,44]. However, the addition of metal ions in general was suitable for aqueous systems while here, the RAFT agents could be appropriate for both aqueous and non-aqueous solutions.

The *M_n_* and dispersity of the extracted poly(CMS) in the ETFE films are shown in Figure 4b,c, respectively. It seemed that the *M_n_* of the poly(CMS) in the ETFE films were almost independent of irradiation dose, with values of about 8000 and 16,000 for grafting with and without RAFT agent, respectively. On the other hand, the dispersity decreased with increase of the irradiation dose, and it also decreased with the addition of RAFT agent. Although the dispersity of the extracted poly(CMS) for the system with RAFT agent was lower than that without RAFT agent, it was quite higher than 1.0, indicating that RAFT polymerization and conventional polymerization occurred simultaneously in the ETFE films during graft polymerization.

The *M_n_* and dispersity of the homopolymer generated in the monomer solution are shown in Figure 4e,f, respectively. In the presence of RAFT agent, the *M_n_* increased linearly with increase in irradiation dose. Furthermore, the dispersity was independent of the irradiation dose, and was very close to 1.0. Consequently, a typical RAFT polymerization occurred in the monomer solution. Similar phenomena were reported for the RAFT-mediated radiation polymerization of vinyl monomers such as acrylamide, acrylic acid, styrene, and their derivatives [13,17]. The added RAFT agents in the monomer solution could effectively trap the radicals generated by irradiation, and the homopolymerization in the monomer solution was controlled at a slow rate. These phenomena could be applied for the synthesis of some monodispersed polymer or oligomer for special applications.

On the contrary, without the addition of RAFT agent, both *M_n_* and dispersity of the homopolymer in the monomer solution increased with increasing irradiation dose. The high values of *M_n_* especially at high irradiation dose may be due to the crosslinking that occurred between the macro-radicals.

It was found that the *M_n_* of the poly(CMS) generated in the ETFE films was quite higher than that of the poly(CMS) homopolymer in the monomer solution. By comparison of Figure 4b,e, the *M_n_* of the poly(CMS) homopolymer in the monomer solution was less than 10,000 while that of the poly(CMS) in the ETFE films was higher than 15,000 in the absence of RAFT agent. The latter was more than 1.5 times higher than the former. When the RAFT agent was added, the difference was more significant; that is, the *M_n_* of the homopolymer in the monomer solution was less than 2000, while that of the poly(CMS) generated in the ETFE films was higher than 6000. The latter was more than three times higher than the former.

Therefore, we can conclude that the polymerizations in the monomer solution and in the ETFE films were different from each other. The graft polymerization in the ETFE films was a solid phase reaction or a heterogeneous reaction, while the homopolymerization in the monomer solution was a homogeneous reaction. In the monomer solution, the generated radicals quickly initiated the polymerization, and the growing chains were easily terminated by the surrounding small molecules or other radicals. In the ETFE films, the generated radicals were fixed in the ETFE crystalline micro-particles or ETFE chains, and the polymerization in the ETFE films proceeded via the grafting front mechanism, where the monomer diffused into the interior of the ETFE films for polymerization [24]. Therefore, the propagating radicals in the solid films were stable compared to those in the monomer solution, resulting in a long lifetime for polymerization and thus a high molecular weight of the poly(CMS) generated in the ETFE films. Thus, the molecular weight of the poly(CMS) formed in the ETFE films is higher than that of the homopolymer formed in the monomer solution.

These results were different from the reports for the styrene-grafted cellulose and the styrene-grafted silica particles, where the graft chains on the substrate and the homopolymer in the monomer solution showed similar *M_n_* and dispersity [29,31,36]. For the styrene-grafted cellulose, the cellulose was well swollen in the monomer solution, and thus the graft polymerization and homopolymerization have a similar reaction environment. On the contrary, for the styrene-grafted silica particles, the monomer cannot diffuse into the particles and only the surface towards the solution were grafted. Therefore, the graft polymerization and homopolymerization also have a similar reaction environment. However, ETFE was not swollen in any monomers or solvents, but the monomer was diffused into the interior of the film according the grafting front mechanism [24], resulting in the different polymerization environments in the ETFE films and in the monomer solution.

The extracted poly(CMS) chains and the homopolymer obtained in the system with RAFT agent showed lower *M_n_* and dispersity than those obtained in the system without RAFT agent, indicating that the RAFT agent really affected the polymerization. Especially, the homopolymerization in the monomer solution with RAFT agent showed characteristics of a typical RAFT polymerization. Furthermore, the poly(CMS) formed in the ETFE films with RAFT agent had a dispersity higher than 1.0 but lower than that of the system without RAFT agent, indicating again that both conventional radical polymerization and RAFT polymerization occurred in the ETFE films. This phenomenon may be caused by the inadequate amount of RAFT agent in the interior of the ETFE films due to its slow diffusion.

### 3.5. Effect of Irradiation Dose Rate

**Figure 5:**
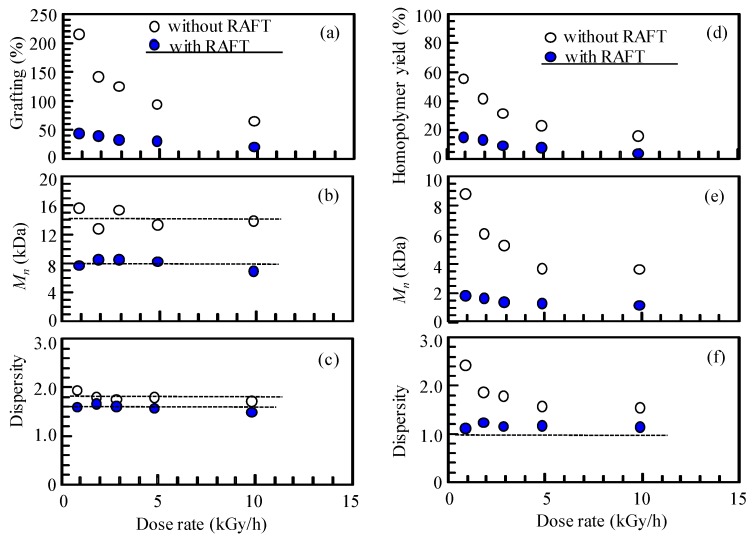
(**a**) Degree of grafting, (**b**) *M_n_*, (**c**) dispersity of the extracted poly(CMS) in the ETFE films, and (**d**) homopolymer yield, (**e**) *M_n_*, (**f**) dispersity of the homopolymer in the monomer solution as a function of the irradiation dose rate. Total irradiation dose, 50 kGy; [RAFT]/[CMS], 0.014.

We have discussed the effects of total irradiation dose on the simultaneous radiation grafting of CMS onto ETFE films at a fixed dose rate of 1.0 kGy/h. In principle, the dose rate is proportional to the generation rate of radicals, and thus strongly affects both graft polymerization and homopolymerization. Figure 5 shows the results of polymerization in the ETFE films (grafting) and in the monomer solution (homopolymerization) as a function of irradiation dose rate.

As shown in Figure 5a,d, all degrees of grafting and homopolymer yield decreased with increasing irradiation dose rate at fixed irradiation dose of 50 kGy. In the absence of RAFT agent, the degree of grafting decreased from 217% to 68%, and the homopolymer yield decreased from 56% to 17% when the irradiation dose rate increased from 1.0 kGy/h to 10 kGy/h. On the contrary, in the presence of RAFT agent, the degree of grafting decreased from 44% to 22%, and the homopolymer yield decreased from 15% to 5.4% when the irradiation dose rate increased in the same range. Therefore, in the range of dose rates studied, both the graft polymerization and homopolymerization were suppressed by addition of the RAFT agent. Lower dose rate during irradiation was favorable for both graft polymerization and homopolymerization. Higher dose rate causes the faster generation of radicals, resulting to higher radical concentration in the system, and thus faster radical consumption due to the coupling or termination reactions, resulting in the lower polymer yield under the same amount of total dose.

When dose rates lower than 3.0 kGy/h were applied, both graft polymerization and homopolymerization decreased drastically in the absence of RAFT agent, while only a slight decrease was observed in the presence of RAFT agent, indicating that the amount of RAFT agent was adequate for trapping the generated radicals at the lower dose rate range. When the dose rate of irradiation further increased, more RAFT agent was needed to turn the active radicals into the dormant state.

However, as shown in Figure 5b,c, the *M_n_* and dispersity of the extracted poly(CMS) from the grated films were almost independent of the irradiation dose rate: about 14,000 and 1.8 in the absence of RAFT agent, and about 8000 and 1.6 in the presence of RAFT agent, respectively. Both *M_n_* and dispersity decreased after addition of the RAFT agent, indicating that both RAFT polymerization and conventional radical polymerization occurred in the ETFE films during grafting.

Figure 5e,f showed the *M_n_* and dispersity of the homopolymerization in the monomer solution as a function of the irradiation dose rate. Without the RAFT agent, *M_n_* decreased from 8800 to 3500 and the dispersity decreased from 2.4 to 1.5 as the irradiation dose rate increased from 1.0 kGy/h to 10 kGy/h. The decreased *M_n_* was due to the high concentration of radicals at high dose rate, while the increased PDI at lower dose rate may be due to the higher viscosity of the grafting medium (or higher homopolymer yield as shown in Figure 5d) that caused the crosslinking under the irradiation. When the RAFT agent was added, the dispersity was close to 1.0 in the whole dose rate range, showing the intrinsic characteristic of RAFT polymerization. In this case, the *M_n_* decreased from 1850 to 1050 when the irradiation does rate increased from 1.0 to 10 kGy/h, and the lower *M_n_* at high irradiation dose rate was due to the lower homopolymer yield in the monomer solution as shown in Figure 5d.

It was found again that the *M_n_* of the poly(CMS) in the ETFE films were quite higher than that of the homopolymer in the corresponding monomer solution. In the absence of RAFT agent, the *M_n_* was about 14,000 for the poly(CMS) generated in the grafted films and around 8800–3500 for the homopolymer generated in the monomer solution, the former being more than 1.6 times higher than the latter. In the presence of the RAFT agent, the difference was more significant. That is, the *M_n_* was about 8000 for the poly(CMS) in the ETFE films and was 1850–1050 for the homopolymers in the monomer solution, the former was more than 4.3 times higher than the latter.

In the interior of the ETFE films, the propagating radicals were fixed in the crystal particles and thus had a longer lifetime for polymerization. Thus, the polymerization in the ETFE films was almost independent of the irradiation dose rate and was slight influenced by the added RAFT agent. On the contrary, the homopolymerization in the monomer solution was a homogeneous reaction. The propagating radicals were easily transferred to other molecules, or terminated by radical coupling. As a result, the polymerization was largely effected by the irradiation dose rate and the addition of RAFT agent.

### 3.6. Effect of Mole Ratio of [RAFT]/[CMS]

In general, the RAFT polymerization is largely affected by the mole ratio of [RAFT]/[CMS]. In an ideal RAFT polymerization, the molecular weight dispersity of the resultant polymer would be 1.0, and the *M_n_* can be calculated using Equation (6).

*M_n_* = 153*P*/100*R* + 221 (6)

where *P* is the homopolymer yield in the monomer solution, *R* is the mole ratio of [RAFT]/[CMS], 153 and 221 are the molecular weights of the CMS monomer and the RAFT agent, respectively. In an ideal RAFT polymerization all the radicals were reacted under the equilibrium state showed in the Scheme 2, formula 2, and the target *M_n_* can be easily controlled by varying the [RAFT]/[CMS] and the degree of polymerization. However, some side reactions, such as radical transfer and termination reactions, would lead to deviation from the ideal RAFT polymerization.

**Figure 6:**
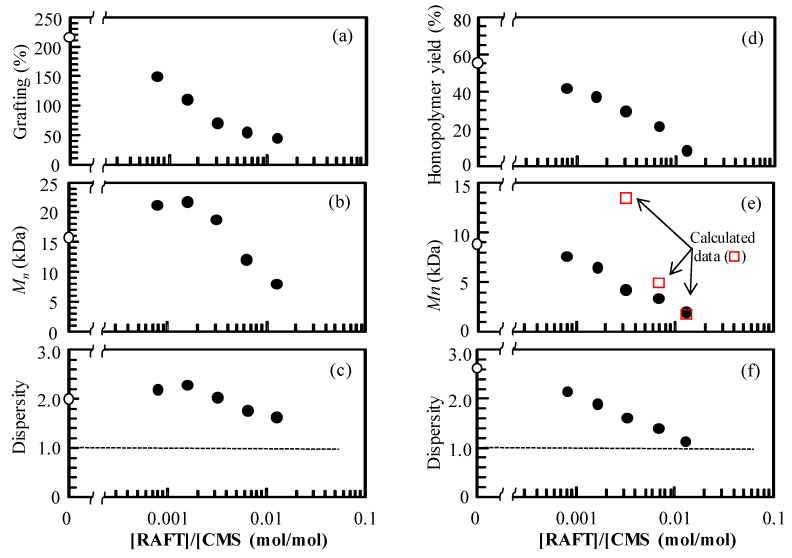
(**a**) Degree of grafting, (**b**) *M_n_*, (**c**) dispersity of the extracted poly(CMS) from grafted ETFE films, and (**d**) homopolymer yield, (**e**) *M_n_*, (**f**) dispersity of the homopolymer in the monomer solution as a function of the mole ratio of [RAFT]/[CMS]. Irradiation dose rate, 1.0 kGy/h; total irradiation dose, 50 kGy; [RAFT]/[CMS], 0.014.

Figure 6 shows the results of grafting and homopolymerization as function of the mole ratio [RAFT]/[CMS]. The total irradiation dose was 50 kGy and the dose rate was 1.0 kGy/h. As seen in Figure 6a,d, both the degrees of grafting and homopolymerization decreased with increasing [RAFT]/[CMS] mole ratio. Similar effects of the RAFT agent concentration on the grafting have reported by Barner et al. [17,18] and Barsbay et al. [45]. Without the RAFT agent, the degree of grafting reached 217%, and the degree of polymerization was 56%. When the mole ratio of [RAFT]/[CMS] increased from 0.00087 to 0.014, the degree of grafting decreased from 150% to 44%, as well as the homopolymer yield decreased from 42% to 14%. Although the RAFT agent suppressed grafting, it also inhibited the homopolymerization in the monomer solution.

Figure 6b,c shows the *M_n_* and dispersity of the poly(CMS) in the ETFE films. Without RAFT agent, the *M_n_* and dispersity were about 16,000 and 2.0, respectively. However, when the RAFT agent was added, both the *M_n_* and dispersity of the poly(CMS) in the ETFE films initially increased slightly and then decreased with increasing [RAFT]/[CMS] mole ratio. It was not well understood why *M_n_* and dispersity increased under low [RAFT]/[CMS] mole ratio. There were two mechanisms for the polymerization in the presence of RAFT agent. One was the general radical polymerization, resulting to poly(CMS) with *M_n_* close to 16,000; the other was the RAFT polymerization. Under the low mole ratio condition, the higher *M_n_* could be obtained for the RAFT polymerization. As a result, the average *M_n_* was higher and the molecular weight was more dispersed (corresponding to a higher dispersity). The maximum *M_n_* and dispersity of the poly(CMS) formed in the ETFE films were observed at the [RAFT]/[CMS] mole ratio close to 0.0017. The high Mn of the poly(CMS) chains in the ETFE films was useful for designing functional materials but the high dispersity was undesirable in many cases. The optimum conditions could be found by adjusting the mole ratio for the target graft materials. Using higher [RAFT]/[CMS] mole ratio, the poly(CMS) in the ETFE films showed a lower dispersity and a lower *M_n_*, indicating that the RAFT polymerization occurred more dominantly in the ETFE films.

On the other hand, as shown in Figure 6e,f, both the *M_n_* and dispersity of the homopolymer in the monomer solution monotonously decreased as the [RAFT]/[CMS] mole ratio increased. The observed trend was in agreement with the characteristics of RAFT polymerization. However, the *M_n_* calculated using Equation (6) were 5000 and 13,500 at [RAFT]/[CMS] mole ratio of 0.007 and 0.0034, while the detected *M_n_* were 3200 and 4000, respectively. The calculated *M_n_* was higher than the experimental *M_n_*. In addition, when the applied [RAFT]/[CMS] mole ratio was lower than 0.007, the obtained dispersity was higher than 1.4. Therefore, in the case of [RAFT]/[CMS] mole ratio lower than 0.007, the small amount of RAFT agent in the monomer solution was inadequate for RAFT polymerization, and the conventional radical polymerization occurred predominantly, resulting to higher dispersity and lower *M_n_* than the theoretical values. On the other hand, when the [RAFT]/[CMS] mole ratio was 0.014, the calculated *M_n_* was in good agreement with the experimental *M_n_*, and the dispersity was close to 1.0, indicating that a typical RAFT polymerization occurred under the [RAFT]/[CMS] mole ratio higher than 0.014 in the simultaneous radiation grafting system.

## 4. Conclusions

The effects of RAFT agent on the simultaneous radiation grafting of CMS onto ETFE films were investigated in detail. Both the graft polymerization and homopolymerization were suppressed by the addition of RAFT agent, resulting in lower degree of grafting and lower homopolymer yield. The non-grafted poly(CMS) in the grafted ETFE films was successfully extracted by xylene at 120 °C. The homopolymer yields were analyzed by both GPC and NMR instruments, and the results were in agreement with each other.

In the presence of RAFT agent, for the polymerization in ETFE films, the conventional radical polymerization took place simultaneously with RAFT polymerization due to the inadequate amount of RAFT agents in the interior of the films. While for the homopolymerization in solution, the ideal RAFT polymerization could be reached under high RAFT agent concentration.

The *M_n_* of the extracted poly(CMS) generated in the grafted ETFE films was several times higher than that of the poly(CMS) generated in the monomer solution. In the presence of RAFT agent, the difference was more considerable. Addition of the RAFT agent decreased the molecular weight dispersity of the poly(CMS) in the ETFE films effectively. In order to develop new functional graft materials, more efforts are needed to produce graft chains with high molecular weight and very narrow molecular weight distribution.
